# Reading Books and Watching Films as a Protective Factor against Suicidal Ideation

**DOI:** 10.3390/ijerph121215032

**Published:** 2015-12-15

**Authors:** Mami Kasahara-Kiritani, Gergö Hadlaczky, Michael Westerlund, Vladimir Carli, Camilla Wasserman, Alan Apter, Judit Balazs, Julio Bobes, Romuald Brunner, Elaine M. McMahon, Doina Cosman, Luca Farkas, Christian Haring, Michael Kaess, Jean-Pierre Kahn, Helen Keeley, Bogdan Nemes, Urša Mars Bitenc, Vita Postuvan, Pilar Saiz, Merike Sisask, Airi Värnik, Marco Sarchiapone, Christina W. Hoven, Danuta Wasserman

**Affiliations:** 1 National Centre for Suicide Research and Prevention of Mental Ill-Health (NASP), Karolinska Institutet, Stockholm 17177, Sweden; Gergo.Hadlaczki@ki.se (G.H.); michael.westerlund@ims.su.se (M.W.); Vladimir.Carli@ki.se (V.C.); Danuta.Wasserman@ki.se (D.W.); 2 Department of Child and Adolescent Psychiatry, New York State Psychiatric Institute, Columbia University, New York, NY 10032, USA; camillawasserman@gmail.com (C.W.); HOVEN@nyspi.columbia.edu (C.W.H.); 3 Feinberg Child Study Centre, Schneider Children’s Medical Centre, Tel Aviv University, Tel Aviv, Israel; eapter@clalit.org.il; 4 Vadaskert Child and Adolescent Psychiatric Hospital, Budapest 1021, Hungary; judit.agnes.balazs@gmail.com (J.B.); lucafarkas@gmail.com (L.F.); 5 Institute of Psychology, Eötvös Loránd University, Budapest 1064, Hungary; 6 Department of Psychiatry, Centro de Investigación Biomédica en Red de Salud Mental, CIBERSAM, University of Oviedo, Oviedo 33004, Spain; bobes@uniovi.es (J.B.); frank@uniovi.es (P.S.); 7 Section for Disorders of Personality Development, Clinic of Child and Adolescent Psychiatry, Centre of Psychosocial Medicine, University of Heidelberg, Heidelberg 69115, Germany; Romuald.Brunner@med.uni-heidelberg.de (R.B.); Michael.Kaess@med.uni-heidelberg.de (M.K.); 8 National Suicide Research Foundation, Cork, Ireland; e.mcmahon@ucc.ie (E.M.M.); mandhhealy@eircom.net (H.K.); 9 Clinical Psychology Department, Iuliu Hatieganu University of Medicine and Pharmacy, Cluj-Napoca 400012, Romania; doina_octaviancosman@hotmail.com (D.C.); nemes_bogdan@yahoo.com (B.N.); 10 State Hospital Hall in Tyrol, tirol-kliniken, Department for Psychiatry and Psychotherapy B, Hall, A-6060, Austria; christian.haring@tirol-kliniken.at; 11 Department of Psychiatry, Centre Hospitalo-Universitaire de Nancy, Université de Lorraine, Nancy, 54500 Vandoeuvre-lès-Nancy, France; jp.kahn@chu-nancy.fr; 12 Slovene Center for Suicide Research, Andrej Marušič Institute, University of Primorska, Koper 6000, Slovenia; ursa.mars@upr.si (U.M.B.); vita.postuvan@upr.si (V.P.); 13 Estonian-Swedish Mental Health & Suicidology Institute, Tallinn 11615, Estonia; sisask.merike@gmail.com (M.S.); varnik.airi@gmail.com (A.V.); 14 Tallinn University, Tallinn 10120, Estonia; 15 Department of Medicine and Health Science, University of Molise, Campobasso 86100, Italy; marco.sarchiapone@me.com; 16 National Institute for Migration and Poverty via San Gallicano 25, Roma 00100, Italy; 17 Department of Epidemiology, Mailman School of Public Health, Columbia University, New York, NY 10032, USA

**Keywords:** Saving and Empowering Young Lives in Europe (SEYLE), adolescent, belonging, mental health, protective factors, suicide

## Abstract

Reading books and watching films were investigated as protective factors for serious suicidal ideation (SSI) in young people with low perceived social belonging. Cross-sectional and longitudinal (12-month) analyses were performed using data from a representative European sample of 3256 students from the “Saving and Empowering Young Lives in Europe” study. Low social belonging was associated to SSI. However, reading books and watching films moderated this association, especially for those with lowest levels of belonging. This was true both at baseline and at 12 months of follow-up analyses. These media may act as sources of social support or mental health literacy and thus reduce the suicide risk constituted by low sense of belonging.

## 1. Introduction

A substantial amount of research has been conducted on the relation between suicidal behavior and consumption of various forms of media, such as newspapers, television, film, music, literature, and, in recent years, different types of communication platforms on the Internet [1]. The vast majority of the research has focused on imitation, or contagion, known as the “Werther effect” [2], *i.e.*, that media coverage of suicide can trigger actual suicidal behavior in vulnerable individuals in the audiences. Some studies have also investigated whether media recommendations on responsible reporting suicide cases have a protective influence, known as the “papageno effect”, on consumers. Niederkrotenthaler, Voracek [3] conducted studies that indicate this type of preventive effect. Not so surprisingly, these types of studies on media-effects in suicide focus mainly on media reporting a *suicide-specific* theme. However, the suicide-related effects of more general media consumption, such as reading books or watching films with non-specific themes, are largely unknown.

Protective factors are less often the focus of research on youth suicides compared to risk factors. Adolescents’ perceptions of belonging have been shown to be related to depression, social rejection, and school problems, as well as to suicidality [4], probably due to a lack of social support. In this study we investigate whether reading books and watching films may constitute a protective factor in adolescents who feel left out. Evidence shows that the amount of reading impacts on social skills as well as community participation [5]. Reading books or watching films may compensate for lacking social support if, for instance, the reader can in some way identify with the narrative, situational factors or protagonists in the stories. Through this identification, readers or watchers may feel less alone, or may learn from these media about coping strategies, help-seeking strategies or perhaps increase their willingness to discuss their problems to health-care professionals or other gate-keepers [6]. We hypothesize that consumption of these forms of media may be protective against suicidal ideation among adolescents experiencing low belonging. This was investigated using a cross-sectional design, as well as a longitudinal design. In the latter, students who had suicidal ideation at baseline were excluded from the analyses and the investigation was thus carried-out on a non-suicidal sample, strengthening the causal aspects of possible findings. 

## 2. Experimental Section 

A representative sample of 3256 adolescents (mean age 15.00 ± 0.51, 60 cases missing; M/F: 1553/1689, 14 cases missing) from 179 randomly selected schools in 11 different European countries were assessed in this study. The sample constitutes the control group of a larger multi-center randomized control trial in the “Saving and Empowering Young Lives in Europe” (SEYLE) study [7]. Ethical approval was obtained from each of the local research ethics committees.

A structured self-report questionnaire was administered to adolescents in the participating schools. Serious suicidal ideation (SSI) was measured using the Paykel suicide ladder [8] and a dichotomous variable was generated. Belonging was measured by the question “You feel you belong to a group” with “rarely or never” (low), “sometimes” (medium), “often or all the time” (high) as possible responses. The students were also asked to list titles of up to three of their favorite books and films they had read or watched in the previous 6 months. The number of books and films listed was used as a predictor variable (number of books read: 0 or 1 = non-readers; 2 or 3 = readers, number of films watched: 0 or 1 = non-watchers, 2 or 3 = watchers). 

The hypotheses were tested using both cross-sectional and longitudinal analyses. SSI at baseline and at 12-month follow-up was used as outcome measure, respectively. In the longitudinal analyses incident SSI was measured to better establish causality and for this purpose those reporting SSI at baseline were excluded. 

Data were stratified according to belonging. Characteristics between students with none-mild (SSI −) and moderate-severe suicidal ideation (SSI +) were compared using χ^2^ tests (for categorical variables) and independent-sample *t*-tests (for continuous variables). Multiple regression analysis was used to investigate the associations between SSI (at baseline and at 12 months) and the amount of books read or films watched. Age (entered as a continuous variable), gender, residence, living condition (living with both parents), and the number of books read or films watched during the previous 6 months were entered separately into the model to adjust for individual characteristics. Because students with SSI at baseline were excluded in the longitudinal analysis (cohort sampling), the estimated odds ratios from the longitudinal analyses were equivalent to incidence rate ratios (IRRs). Analyses were carried out in Statistical Package for Social Sciences (SPSS version 22.0). 

## 3. Results and Discussion

### 3.1. Results

Description of study population is shown in Table 1. Students without SSI at baseline (M/F: 1429/1539) read significantly more books (*n* = 1.83 ± 1.27, *p* < 0.01, *t* = 3.838, degrees of freedom: *df* = 3211) or watched more films (*n* = 2.47 ± 0.99, *p* < 0.01, *t* = 3.224, *df* = 259.628) than students with SSI (books: *n* = 1.50 ± 1.28, films: *n* = 2.22 ± 1.14). Ratios of belonging were significantly different between students with and without SSI (*p* < 0.01, χ*^2^* = 32, *df* = 2). Among the students without SSI at baseline, 62 (2.1%) had SSI at 12 months follow up.

**Table 1:** Description of study population.

Studied Variables	SSI at Baseline		*p*-Value
–	SSI −	SSI +	–
*n* (%)	2980 (92.7)	233 (7.2)	–
Age mean ± SD	14.87 ± 0.92	14.95 ± 0.968	0.24
Gender male (%)	1429 (93.8)	95 (6.2)	0.02
Female (%)	1539 (73.9)	137 (8.2)	–
Number of books mean ± SD	1.83 ± 1.27	1.50 ± 1.28	0.00
Number of films mean ± SD	2.47 ± 0.99	2.22 ± 1.14	0.00
Belonging Low (%)	261 (8.9)	37 (16.5)	0.00
Middle (%)	730 (25.0)	80 (35.7)	–
High (%)	1933 (66.1)	107 (47.8)	–
Worthlessness High (%)	12 (0.4)	18 (7.8)	0.00
Low (%)	2537 (85.7)	102 (44.2)	–
Belonging and worthlessness: % within SSI +/−		–	–

**Figure 1:**
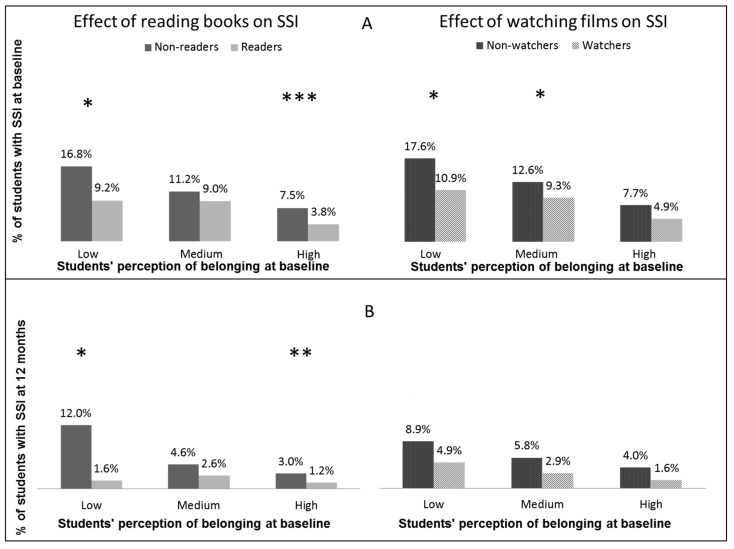
(**A**) Results of the cross sectional analysis showing the proportion of students with SSI at baseline for each level of the variable belonging. (**B**) Results from the longitudinal analysis showing the proportion of students with incident suicidal ideation at 12 months based on their levels of belonging at baseline (students reporting SSI at baseline were excluded from this analysis). In this figure, * *p* < 0.05, ** *p* < 0.01, *** *p* < 0.001.

The numbers of students reporting SSI were larger for non-readers or non-watchers than that for readers or watchers at all levels of belonging (see Figure 1). The rate of students with SSI was significantly lowered with increasing numbers of books reported by students with low or high levels of belonging (Odds ratio: OR = 0.73, 95% Confidence interval: 95% CI = 0.54–0.98, *p* = 0.04 for low level of belonging, OR = 0.72, 95% CI = 0.61–0.85, *p* < 0.01 for high level of belonging). The rate of SSI was significantly lowered with increasing number of films reported among students with low or medium levels of belonging (OR = 0.73, 95% CI = 0.54–0.98, *p* = 0.04, OR = 0.76, 95% CI = 0.61–0.95, *p* = 0.02, respectively). At 12 months, IRR of SSI in students with low or high belonging at baseline were significantly lowered with increasing number of books read (IRR = 0.51, 95% CI = 0.29–0.92, *p* = 0.024, IRR = 0.62, 95% CI = 0.45–0.85, *p* < 0.01, respectively). 

### 3.2. Discussion 

The results confirm previous findings identifying perceptions of belonging as predictor of suicidality [4,9]. Moreover, the results also support the hypothesis that reading books or watching films act as protective factors in terms of SSI in students who have a low sense of belonging. This was the case both at the baseline and at a 12-month follow-up (Figure 1). 

The contribution of media to behavioral change can be explained in various ways [10]. Although research regarding media’s role in suicidality has up to date primarily focused on media with suicide related content, our findings suggests that other aspects of the media should also be considered in terms of suicide prevention. It seems plausible that reading books or watching films, may to an extent, compensate for lacking social contact. It is possible that these activities act as a protective factor against suicidal ideation by providing social support [11,12]: Adolescents may benefit from healthy peer-relationship, gaining role-models, emotional or motivational support, a sense of belonging, *etc.*, through affiliation and identification. These same processes (*i.e.*, affiliation and identification) may also be targeting positive role-models in book/movie characters [13]. Other plausible explanations may be that books and films work as an escape, or retreat from daily stressors [14], and serve as a tool to develop better stress coping skills, through an increase in mental health literacy, by example how book and film characters handle different problems. 

Future research may want to explore motivations for the students to engage in reading or watching films to further support the potential theories proposed in this article, e.g., was the motivator a form of avoidance/escape, or a desire to “belong”; the content of the media itself, which was beyond the scope of this study, may also lend some light to provide explanation. The specific mechanism behind the effects of reading books and watching films, in relation to suicidality, needs further investigations. More studies are required to specify the target of belonging.

All valuables were measured by one-item scales, which were not evaluated in terms of validity or reliability. Additionally, using more detailed measures, which are more inquisitive as to the *nature* of media consumed, may give a clearer picture on different aspects of the reading or watching effects on loneliness. Another limitation is that the results may be biased by other potential intervening variables such as early maltreatment [15], even if other possible cofounders such as age, gender, residence, and living condition were included in the regression model. 

## 4. Conclusions

Books and films may act as sources of social support or mental health literacy and thus reduce the suicide risk constituted by low sense of belonging. Recommendation that students with a low sense of belonging, who are at risk of suicidal behavior, should read books and/or watch films may have preventive effects.
